# mSphere of Influence: Retreading old ground—the beauty in multi-omic data sets

**DOI:** 10.1128/msphere.00131-24

**Published:** 2024-05-15

**Authors:** Ethan L. Morgan

**Affiliations:** 1 School of Life Sciences, University of Sussex, Brighton, United Kingdom; Virginia Commonwealth University, Richmond, Virginia, USA

**Keywords:** HPV, HNSCC, cervical cancer, ubiquitination

## Abstract

Ethan L. Morgan works on human papillomaviruses (HPVs), with a specific interest in identifying how HPV induces tumor formation. In this mSphere of Influence article, he reflects on how three papers influenced him. “Comprehensive genomic characterization of head and neck squamous cell carcinomas” (The Cancer Genome Atlas Network, Nature 517:576–582, 2015, https://doi.org/10.1038/nature14129) and “Integrated genomic and molecular characterization of cervical cancer” (The Cancer Genome Atlas Network, Nature 543: 378–384, 2017, https://doi.org/10.1038/nature21386) showed him the power behind comprehensive multi-omic analyses to understand disease biology, while “Human papillomavirus E7 oncoprotein targets RNF168 to hijack the host DNA damage response” (J. Sitz et al., Proc Natl Acad Sci U S A 116:19552–19562, 2019, https://doi.org/10.1073/pnas.1906102116) reinforced how this can be used to undercover potential new drug targets in HPV-associated disease.

## COMMENTARY

I distinctly remember my first introduction to virology in the second year of my undergraduate degree. The simplicity of how small particles can cause such immense damage really resonated with me and this, coupled with other lectures on anti-viral signaling pathways, sparked my desire to pursue a PhD in virology (I still remember drawing out the innate immune signaling pathway in a third-year exam!). The study of viruses has always played an important role in uncovering new biology, from the identification of tumor suppressor proteins such as p53 and pRb (1, 2), to the demonstration of molecular evolution in driving the “arms race” between viruses and the host (3). Understanding virus-host interactions can lead to the identification of ways to treat viral infection and disease. In these papers, The Cancer Genome Atlas Network utilizes mult-omics approaches to understand the genomic, transcriptomic, and proteomic landscape of head and neck squamous cell carcinoma (HNSCC) and cervical cancer, two cancers associated with HPV infection. The data from these papers were subsequently used by Sitz et al. to uncover a novel mechanism of viral replication that may contribute to the genomic stability observed in HPV-associated cancers.

HPV is the leading cause of viral-induced carcinoma, causing around half of all virus-associated cancers worldwide. Work from Harald Zur Hausen (and colleagues), which led to him being awarded the Nobel Prize in Physiology or Medicine, established that HPV was the primary causal factor leading to the development of cervical cancer. Subsequent work has identified at least five other cancers caused by HPV to varying prevalences: anal, vaginal, vulva, penile, and cancers of the head and neck region. While previous studies demonstrated that the HPV oncoproteins E6 and E7 are the primary driver of oncogenesis, the impact of different types of HPV and their role in cancer development, and the genomic alterations observed in HPV+ cancers were unclear. The Cancer Genome Atlas (TCGA) project was established to catalog the genomic alterations observed in multiple cancers through the use of high-throughput, multi-omics techniques. In 2015 and 2017, TCGA released their characterization of HNSCC and cervical cancer, based on an analysis of 279 and 228 patients, respectively (4, 5). The data gained from this study provided great insight into the biological difference between HPV+ and HPV− cancers at similar anatomical sites, identifying differential integration patterns observed between different HPV types in cervical cancer, and novel deletions in the *TRAF3* gene in HPV+ HNSCC, suggesting aberrant activation of NFκB signaling in this subset of HNSCC. These comprehensive genomic studies changed our understanding of HPV-mediated oncogenesis and highlighted novel therapeutic targets that could be clinically beneficial in these cancers. Furthermore, they illustrated the power of these high-throughput, multi-omic approaches and how they offer essential insights into disease biology.

Following my PhD studies, which were focused on how HPV manipulates cell signaling networks to promote oncogenesis, I was searching for a new direction to pursue my research niche. A conversation with my PhD mentor Andrew Macdonald led me to look at protein ubiquitination, a vital post-translation modification that regulates almost all biological processes. My journey in this new field started using the resources available through the TCGA data sets uncovered in these two manuscripts. Access to this data has been made highly accessible through the cBioPortal, an open-access resource for interactive exploration of multiple cancer databases, including all of the TCGA data (6). This made exploring all of this genomic and transcriptomic data incredibly easy, even for someone with limited bioinformatic experience (like me!).

This approach is exemplified by Sitz et al. (7), who investigated the occurrence of large foci of 53BP1 previously observed in HPV+ tumors (8). This study expands on this observation and establishes that HPV-mediated hijacking of RNF168 is a critical inducer of HPV-induced genomic instability that contributes to oncogenesis. Specifically, they demonstrate that RNF168 is required for viral replication and that HPV E7 binds to RNF168 and impairs its ability to function at double-strand breaks (DSBs), promoting genomic instability. The TCGA papers provide essential data demonstrating the increased expression of RNF168 in HPV+ cancers, which provides important insight into how HPV proteins may subvert DSB signaling and repair pathways, potentially highlighting novel therapeutic avenues in these cancers. In a broad context, these three papers have taught me invaluable lessons that I carry with me in my current research. Large-scale, high-throughput experiments can deliver exceptional levels of novel insight into biological systems; however, informative studies can be performed by analyzing data that is already out there, as achieved in Sitz et al. Our recent studies have followed a similar approach, demonstrating how the resources provided by the TCGA studies can offer new insight into how HPV promotes oncogenesis (9) and provide vital context to our *in vitro* studies (10).

These papers have really focused my thinking when it comes to research and reiterated that the most important thing to remember is: what is the question we want to answer? As scientists, we constantly face the pressure of “publish or perish” and getting our next grant funded. Utilizing the vast amounts of data available from these large-scale, multi-omics data sets ensures that we use this funding in the best way possible. There is an incredible amount of data out there; the trick is to ask the right questions and design the appropriate experiments to exploit this wealth of data for maximum impact.
